# HKDC1 in Cancer: Mechanisms, Clinical Applications, and Future

**DOI:** 10.7150/jca.116277

**Published:** 2025-08-22

**Authors:** Xia Luo, Mingjing Peng, Zhan Wang, Nayiyuan Wu, Ying Wang

**Affiliations:** 1 Department of the Central Laboratory, The Affiliated Cancer Hospital of Xiangya School of Medicine, Central South University/Hunan Cancer Hospital, Changsha 410013, Hunan, China.; 2 Lung Cancer and Gastrointestinal Unit, Department of Medical Oncology, The Affiliated Cancer Hospital of Xiangya School of Medicine, Central South University /Hunan Cancer Hospital, Changsha 410031, China.

**Keywords:** HKDC1, cancer, metabolism, biomarker, therapeutic target

## Abstract

Cancer incidence and mortality rates are on the rise, with ovarian cancer being a significant concern globally. HKDC1 is identified as a crucial protein involved in cancer metabolism, particularly in lung, liver, colorectal, and gastric cancers. Its upregulation correlates with poor clinical outcomes and promotes tumor progression through various mechanisms, including enhanced glycolysis and immune evasion. The exploration of HKDC1's role in cancer offers potential for new therapeutic strategies and biomarkers in cancer treatment.

## 1. Introduction

Cancer encompasses a spectrum of malignant diseases posing a significant global health burden. Annually increasing incidence and mortality rates, driven by demographic shifts including population growth and aging, impose substantial medical and economic strain on healthcare infrastructures. According to the Global Cancer observatory, ovarian cancer (OC) ranks as the sixth leading cause of cancer mortality among women in the United States and the eighth worldwide 1. Within China, an estimated 41,436 new ovarian cancer diagnoses and 20,065 related fatalities occurred in 2024^2^.

While cancer mortality has continued a downward trajectory through 2021, averting over 4 million deaths since 1991 due to reduced tobacco use, earlier detection of specific malignancies, and enhanced therapeutic options in both adjuvant and metastatic settings, these gains face challenge from the increasing incidence of six major malignancies. This epidemic not only affects individuals but also places a significant strain on healthcare systems globally, necessitating efficient allocation of medical resources and innovative management strategies 3. The escalating cancer burden underscores a mediacl imperative for effective prevention, early detection, and novel therapeutics to improve patient outcomes and mitigate healthcare strain. Despite the development and clinical deployment of targeted agents inhibiting specific molecular pathways, cancer remains a persistent therapeutic challenge 4. Consequently, the identification of novel therapeutic targets constitutes a critical and ongoing priority in oncology research.

HKDC1 (Hexokinase Domain Containing 1), localized on chromosome 10 adjacent to HK1, encodes a 100-kDa protein exhibiting approximately 70% sequence similarity with HK1^5^. It demonstrates differential expression across multiple tissues, including brain, colon, kidney, liver and lung, among others 6. HKDC1 recently emerged as associated with gestational glucose levels during 2-hour glucose tolerance assessments performed at 28 weeks of gestation in a genome-wide association study 7. This protein has garnered considerable attention due to its involvement in cellular metabolism, especially in glucose regulation. HKDC1 share structural homology with other hexokinases, essential enzymes governing glucose metabolism 8, yet possesses unique features suggesting distinct regulatory functions, potentially influencing metabolic pathways in cancer cells 9. In addition, HKDC1 significantly contributes to cellular metabolism, where dysregulation is a hallmark of tumorigenesis. Cancer cells frequently exhibit heightened glycolytic flux, known as the Warburg effect, facilitating survival in hypoxic microenvironments and supporting rapid proliferation 8. This metabolic reprogramming is fundamental to cancer biology and represents a promising avenue for identifying therapeutic targets.

Researching HKDC1 holds significant importance given its dual potential as a biomarker and therapeutic target across diverse malignancies. Elucidating its function in cancer metabolism could yield critical mechanistic insights, fostering innovative therapeutic strategies, particularly for malignancies with dysregulated metabolism. Therefore, this review aims to synthesize current knowledge regarding HKDC1, including its structural attributes and its correlation with cancer metabolism reprogramming, thereby underscoring its research significance and clinical translational potential.

## 2. Role of HKDC1 in Different Types of Cancer (Table 1)

### 2.1 HKDC1 in lung cancer

HKDC1 demonstrates significant upregulation in lung adenocarcinoma, where it critically promotes oncogenic processes including cell proliferation, migration, invasion, epithelial-mesenchymal transition (EMT), and tumorigenicity while enhancing glycolysis through the AMPK/mTOR signaling pathway. Consistent findings from multiple studies confirm HKDC1 overexpression across lung cancer subtypes 9-13. Mechanistically, HKDC1 enhances glycolytic flux through modulation of the AMPK/mTOR signaling pathway, thereby supporting cancer cell metabolism and growth 9. Additionally, HKDC1 functions as a key regulator of glycolysis and correlates with adverse clinical outcomes in lung squamous cell carcinoma (LUSC). It has been validated as an independent prognostic biomarker, both individually and as part of a four-gene glycolytic signature, highlighting its potential as a therapeutic target in pulmonary malignancies 10.

### 2.2 HKDC1 in liver cancer

HKDC1 is significantly overexpressed in hepatocellular carcinoma (HCC) compared to non-cancerous tissues and is associated with poor overall survival rates in patients. HKDC1 has been linked to various mechanisms that promote cancer progression 14. HKDC1 exhibits marked overexpression both in hepatocellular carcinoma (HCC) 15 and cholangiocarcinoma (CCA) compared to non-neoplastic tissues 16, correlating with diminished overall survival (OS) in HCC patients 15. Studies suggest that HKDC1 promotes tumor immune evasion through cytoskeletal-mediated STAT1 activation and downstream PD-L1 expression, which help cancer cells immune escape 14. Critically, HKDC1 sustains liver cancer progression via essential interactions with mitochondria; disruption of this interface induces mitochondrial dysfunction. Given its tumor-selective expression profile—minimal in normal hepatocytes versus abundant in malignancies, targeting HKDC1, particularly its association with the mitochondria, could offer a promising therapeutic strategy with high molecular specificity^16^ ^17^. Additional evidence from hepatic overexpression models reveals HKDC1's role in augmenting hepatocyte size and proliferative capacity, further implicating it in hepatocarcinogenesis 7. These findings establish HKDC1's role in promoting tumor immune evasion. Its crucial interaction with mitochondria highlights HKDC1 as a promising therapeutic target in liver cancer treatment.

### 2.3 HKDC1 in colorectal cancer

In colorectal carcinoma, HNF1ɑ enhances HKDC1 transcriptional activity, activating the AKT/AMPK signaling pathway crucial for cancer cell metabolism 18. CircVMP1 upregulates HKDC1, driving increased glycolysis and disease progression while contributing to chemoresistance, compromising therapeutic efficacy and underscoring its role in tumor aggressiveness 19. This chemoresistant effect has been further validated, highlighting challenges in therapeutic strategies for colorectal cancer patients 20. Furthermore, HKDC1 exhibits a reciprocal relationship with the circadian regulator BMAL1: BMAL1 knockdown elevates HKDC1 expression and metabolic flux, whereas HKDC1 suppression increases BMAL1 and reduces glycolysis 21, positioning HKDC1 as a potential biomarker for therapy response.

### 2.4 HKDC1 in gastric cancer

HKDC1 is remarkably expressed in Gastric Cancer (GC) tissues and is related to shorter survival rates, suggesting a potential association with tumor progression 22. It collaborates with G3BP1 to stabilize the PRKDC transcript, forming an HKDC1/G3BP1-PRKDC axis that reprograms lipid metabolism to facilitate metastasis and cisplatin resistance—offering a targeted strategy for HKDC1-overexpressing GC 23. Clinically, elevated HKDC1 associates with adverse outcomes, supporting its utility as a diagnostic and prognostic biomarker 22,24. Functionally, suppressing HKDC1 attenuates malignant phenotypes including proliferation, invasion, chemoresistance, glycolytic flux, and EMT *in vitro* and *in vivo* 22,24,25. It forms a regulatory axis with G3BP1 that enhances PRKDC transcript stability, promoting metastasis and chemoresistance through lipid metabolism reprogramming, making it a potential diagnostic and therapeutic target in gastric cancer.

### 2.5 Research on HKDC1 in other cancer types

Beyond the aforementioned cancers, HKDC1 contributes to the pathogenesis of several additional tumors. HKDC1 dysregulation extends to pancreatic adenocarcinoma, where prominent expression enhances proliferation, migration, invasion, and glycolytic flux while suppressing apoptosis and modulating immune infiltration 26. In endometrial cancer under hyperglycemic conditions, the miR-876-5p/HKDC1 axis drives cell proliferation and migration, underlying metabolic adaptation in diabetic patients 27. HKDC1 elevation is observed in HPV8 E7-transduced normal human epidermal keratinocytes 28. Extranodal NK/T-cell lymphoma (ENKTL) exhibits marked HKDC1 upregulation; its depletion disrupts vascular endothelial growth factor 1 (VDAC1) interaction, inducing mitochondrial dysfunction and reactive oxygen species (ROS) overproduction that suppress EBV replication and P-glycoprotein expression. HKDC1 is prominently expressed in both breast cancer cells and clinical tumor specimens. Localized to the mitochondrial membrane, it regulates permeability transition pore opening through interaction with VDAC1, thereby influencing glucose uptake and cellular proliferation. Both *in vitro* and *in vivo*, HKDC1 overexpression accelerates proliferation, metastasis, and tumor growth while reducing survival 27,28. These findings across different cancer types suggest that HKDC1 is implicated in various cancers, enhancing proliferation, migration, and invasion in pancreatic adenocarcinoma, endometrial cancer, and ENKTL, while also influencing metabolic regulation and immune infiltration. Its expression on the mitochondrial membrane and interaction with VDAC1 affect glucose uptake and tumor growth, suggesting HKDC1 as a potential therapeutic target across malignancies.

## 3. Regulatory Mechanisms of HKDC1 in Human Cancers

**Figure 1:**
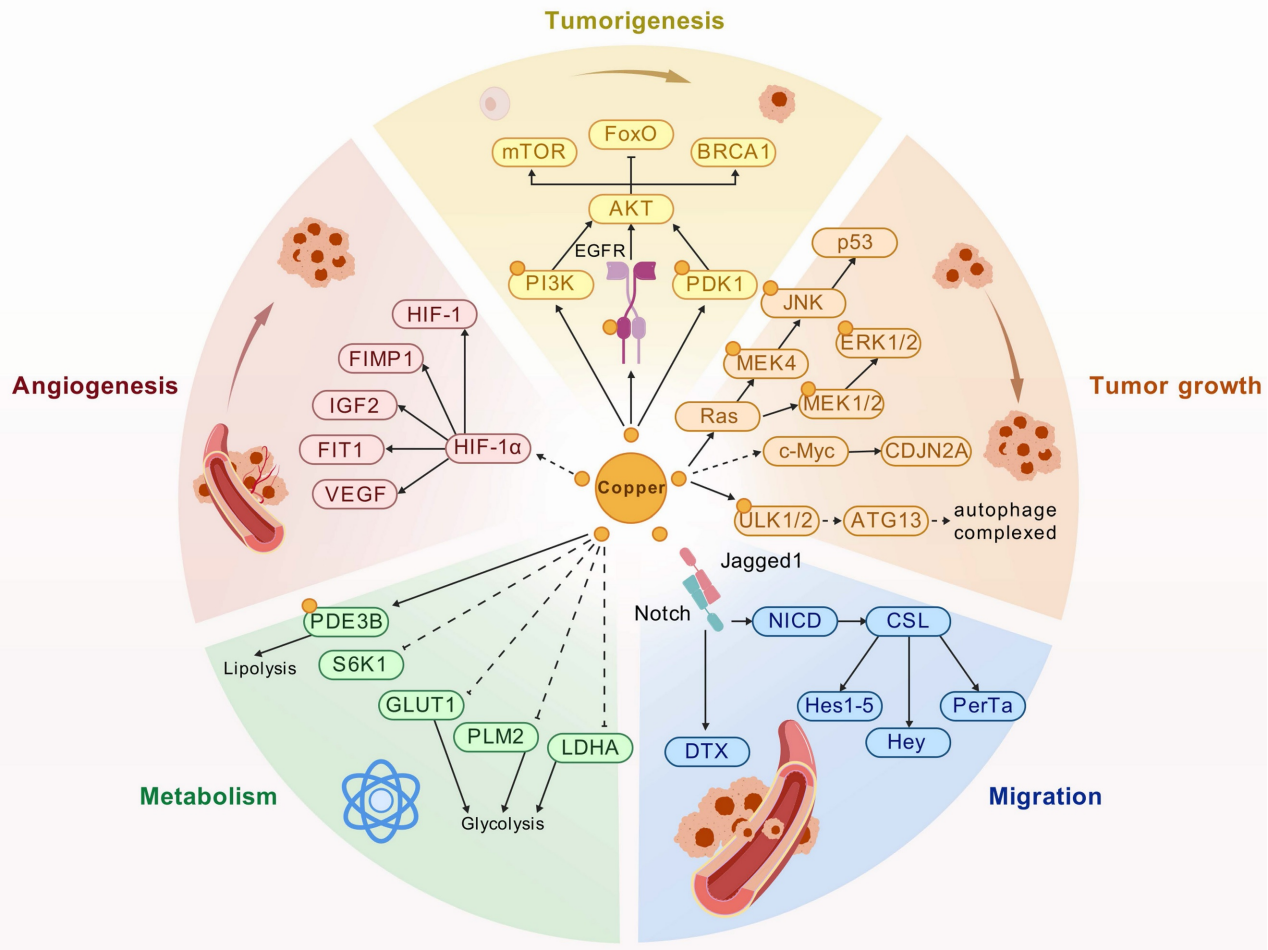
Schematic diagram of regulatory mechanism of HKDC1 in cancers. Created with BioGDP.com 33.

HKDC1 demonstrates consistent overexpression across malignancies, functioning as a potent oncoprotein that orchestrates proliferation, migration, invasion, glycolysis, EMT, and tumorigenicity (Table 1). Its mechanistic influence involves multifaceted regulatory networks (Fig. 1).

### 3.1 Regulating tumor cell metabolism

HKDC1 serves as a critical regulator of tumor metabolism, particularly through glycolytic pathway activation. Its upregulation enhances glycolytic flux and accelerates progression in gastric, hepatocellular, pancreatic, and lung adenocarcinomas. For instance, a study showed that HKDC1 promotes tumorigenesis and glycolytic metabolism in lung adenocarcinoma by regulating the AMPK/mTOR signaling pathway, suggesting a direct link between HKDC1 expression and metabolic reprogramming of cancer cells 9. In gastric cancer, HKDC1 overexpression correlates with chemoresistance and aggressive phenotypes^25^, while its ablation increased oxygen consumption and reduced glycolytic protein expression while inhibiting glucose uptake, lactate synthesis, ATP levels, and extracellular acidification ratio^24^. The research indicated that the absence of HKDC1 markedly enhances glucose uptake and utilization; cells lacking of HKDC1 demonstrate increased glucose consumption, motivating the pentose phosphate (PPP) and hexosamine biosynthesis (HBP) pathways while reducing TCA cycle flux^17^. Hepatocellular carcinoma progression involves HKDC1-mediated TCA cycle modulation 16, and pancreatic cancer studies confirm reduced glucose consumption and lactate output following HKDC1 knockdown in SW1990 cells 26. This metabolic variation not only facilitates rapid proliferation but also supports in the survival of cancer cells under metabolic stress, underscoring HKDC1 as a promising therapeutic target for metabolic intervention in cancer treatment. It indicating that HKDC1 is a promising therapeutic target for metabolic intervention in cancer treatment.

### 3.2 HKDC1 in cancer tumorigenesis

HKDC1 drives key oncogenic processes, inlcuding cell proliferation, migration, invasion, apoptosis and EMT in various cancer types. Elevated HKDC1 correlates with increased cell division and diminished apoptotic activity, crucial for tumor growth and metastasis. In gastric cancer, HKDC1 silencing markedly reduces cellular proliferation and glycolysis, highlighting the essential role of HKDC1 in maintaining the proliferative capacity of these cells 22-25. Furthermore, HKDC1 has been shown to interact with several signaling pathways that regulate cell survival and death. In lung adenocarcinoma, HKDC1 may influence the AMPK/mTOR signaling pathway to enhance proliferation, migration, invasion, glycolysis, EMT, and tumorigenicity 9. imilarly, HKDC1 knockdown hindered cellular proliferation and migration in HCC cells *in vitro*, while in pancreatic cancer it promotes proliferation, migration, and invasion while inhibiting apoptosis 15,16,26. The ability of HKDC1 to modulate these essential cellular processes indicated its potential as a prognostic indicator and as a promising target for therapeutic strategies aimed at reactivating apoptosis in tumor cells.

### 3.3 HKDC1in tumor microenvironment

HKDC1 significantly influences the tumor microenvironment (TME), a critical determinant of cancer progression. Emerging evidence indicates that HKDC1 not only acts directly on cancer cells but also modulates the TME through immune cell infiltration and metabolic crosstalk. In pancreatic adenocarcinoma, HKDC1 overexpression can suppress anti-tumor immunity by reducing immune cell infiltration, correlating with poorer patient outcomes 26. Hepatocellular carcinoma studies demonstrate that aberrant HKDC1 expression promotes immune evasion by inducing CD8+ T-cell exhaustion and facilitating PD-L1-mediated immunosuppression 14. Furthermore, under hyperglycemic conditions, the HOXC-AS2/miR-876-5p/HKDC1 axis drives endometrial cancer progression, illustrating how HKDC1 interacts with environmental factors to promote tumorigenesis 27. These findings position HKDC1 as a dual modulator of tumor cell biology and microenvironmental dynamics, suggesting its targeting could yield multifaceted therapeutic benefits.

## 4. The Correlation between HKDC1 and Cancer Metastasis

### 4.1 Regulation of HKDC1 in metastasis-related genes

HKDC1 is a newly identified metabolic regulatory element, whose upregulation is tightly linked to metastatic competence across multiple cancers. Liqin Zhu *et al.* found that its prometastatic role in facilitating secondary site colonization, particularly in pulmonary metastases 16. By reprogramming glycolytic and lipid metabolic pathways, HKDC1 enhances invasiveness and chemoresistance., for example, in gastric cancer, where it drives cisplatin resistance alongside glycolysis up-regulation 23. Hepatocellular carcinoma models reveal that HKDC1 suppression downregulates β-Catenin and c-Myc expression, indicating that reduce the level of HKDC1 may inhibit cellular proliferation and migration by suppressing the Wnt/β-catenin signaling pathway in HCC 15. In addition, colorectal cancer progression involves HKDC1-mediated AKT/AMPK signaling pathway activation via HNF1α transcriptional regulation 18. In gastric cancer, HKDC1 promotes EMT, further enabling metastatic dissemination 25. This regulatory mechanism reinforces that its centrality in metastasis-related gene regulation and underscores its value as a target to impede tumor spread by affecting cellular metabolism and various signaling pathways.

### 4.2 The influence of HKDC1 on cancer cell migration and invasion

HKDC1 enhances the migration and invasion capacities of cancer cells by reprogramming their metabolic landscape. In pancreatic adenocarcinoma, the upregulation of HKDC1 is closely associated with cell proliferation, migration, and glycolysis, as well as immune infiltration, further suggesting its critical role in the tumor microenvironment 26. Similarly, in lung adenocarcinoma, HKDC1 promotes the tumorigenesis and bolsters glycolytic flux via the AMPK/mTOR signaling pathway, thereby facilitating invasiveness 9. Additionally, overexpression of HKDC1 significantly increased cell migration and invasion in gastric cancer cell lines (SGC-7901, SNU) 23, whereas its silencing by Deng Zhao *et al.* substantially reversed this phenomenon 22. Furthermore, in breast cancer, HKDC1 governs SREBP1-mediated metabolic programs that support tumor growth and metastatic dissemination 29. Collectively, these studies position HKDC1 as both a biomarker of metastatic potential and a promising therapeutic target to curb cancer cell migration and invasion.

## 5. Potential of HKDC1 as a Biomarker

HKDC1 is identified as a promising biomarker for early cancer diagnosis, with significantly elevated expression across multiple malignancies, including gastric, lung, hepatocellular, and pancreatic cancers, correlating with tumor growth and glycolytic activity. Additionally, HKDC1 serves as a prognostic indicator, with higher levels associated with poor outcomes and overall survival in cancers such as colorectal and lung squamous cell carcinoma, influencing tumor-immune interactions and aiding in personalized therapeutic strategies.

### 5.1 Application of HKDC1 in early cancer diagnosis

HKDC1 has emerged as a compelling biomarker for the early diagnosis of various cancers in recent years 22,26,30. Its central role in glucose metabolism, particularly within the altered metabolic pathways associated with cancer, establishing it as a significant factor in tumorigenesis. Research have demonstrated that HKDC1 expression is significantly increased in various cancers, such as gastric, lung, hepatocellular, and pancreatic cancers, which indicating its potential for early identification (Table 1). For example, strong correlations between HKDC1 expression and the growth and glycolytic activity of pancreatic adenocarcinoma cells support its role in the identifying early metabolic alterations 26. Additionally, HKDC1 enhances diagnostic specificity in gastric cancer by discriminating malignant tissue and augmenting existing diagnostic modalities 22. Integration into multi-omics strategies could further exploit its metabolic functions and pan-cancer expression patterns to refine detection strategies 30. Overall, the evidence supports the idea that HKDC1's role in glucose metabolism, along with its correlation with tumor growth and glycolytic activity, underscores its potential for enhancing diagnostic capabilities. Furthermore, it can be integrated into multi-omics strategies for improved detection across various cancer types. HKDC1 could serve as a valuable biomarker for early cancer diagnosis, potentially facilitating its incorporation into routine clinical evaluations.

### 5.2 HKDC1 as a prognostic indicator in cancer

In recent years, cancer investigations have increasingly focused on the prognostic significance of HKDC1, showing that its expression levels are closely associated with patient outcomes (Table 2). Increased HKDC1 levels have been related to poor outcomes in various cancers, such as colorectal and lung squamous cell carcinoma. For example, in lung squamous cell carcinoma, a glycolysis-based three-gene signature inclusive of HKDC1 robustly predicts overall survival 10-12. Moreover, its impact on the immune microenvironment, evident in pancreatic adenocarcinoma immune-infiltration patterns, further augments its prognostic accuracy 26. Another study found that HKDC1 expression continued to be a significant factor affecting overall survival (OS) 9,31. Hepatocellular carcinoma studies establish significant correlations between HKDC1 expression and tumor burden 16,17. Furthermore, the proteogenomic characterization of intrahepatic cholangiocarcinoma has identified HKDC1 as a discriminator of clinically relevant subgroups, guiding tailored therapeutic strategies 32. In conclusion, the accumulating evidence of HKDC1's association with immune cell infiltration and tumor size further underscores its role in overall survival and personalized therapeutic strategies in conditions like pancreatic adenocarcinoma and hepatocellular carcinoma.

## 6. Conclusion

HKDC1 demonstrates consistent overexpression across malignancies, functioning as a key oncogene, driving the growth of tumor cells, migration, invasion, glycolysis, EMT, and tumor formation. By orchestrating signaling cascades, such as AMPK/mTOR, Wnt/β-catenin, and AKT/AMPK, and reprogramming metabolic pathways including glycolysis, lipid metabolism, and the tricarboxylic acid cycle, HKDC1 enhances tumor aggressiveness and chemoresistance. Furthermore, HKDC1 affects the tumor microenvironment by inhibiting immune cell infiltration and promoting tumor immune evasion, such as enhancing CD8+ T cell exhaustion and PD-L1 mediated immune escape. This effect further accelerates tumor progression and metastasis. These findings suggest that HKDC1 is an important regulatory factor in tumor metabolism and biological behavior, serving as a key node in tumor immune regulation.

In clinical settings, elevated HKDC1 is closely associated with poor prognosis across multiple cancers, underscoring its dual utility as a diagnostic and prognostic biomarker. Its levels associate with tumor burden, diminished overall survival, and chemotherapy resistance, suggesting its clinical usefulness in guiding personalized treatment strategies. Moreover, therapeutic approaches targeting HKDC1 are expected to improve cancer treatment outcomes by intervening in tumor metabolism and enhancing the tumor immune microenvironment, making it an important focus for future cancer metabolic interventions and immunotherapy. HKDC1 expression aids in distinguishing malignant tissues and improves diagnostic accuracy and prognostic predictions by integrating multi-omics data. In summary, HKDC1 is a key player in cancer metabolic regulation and an important target for future cancer treatments and personalized medicine. It deepens our understanding of cancer metabolic mechanisms and offers new paths for accurate cancer diagnosis and treatment.

Future research should focus on elucidating HKDC1's broader interactions within tumor biological networks and its crosstalk with complementary metabolic pathways. Additionally, it is essential to investigate its biomarker utility for early detection and prognosis, as this could provide vital insights for risk stratification to inform therapeutic personalization. Such efforts will advance our understanding of HKDC1's biological functions, we may unlock novel metabolic and immunotherapeutic approaches that transform cancer diagnosis and therapy in the future.

## Figures and Tables

**Table 1:** Functional characterization of HKDC1 in various cancers

Cancer type	Expression	Role	Biological function	Related genes	References
Lung adenocarcinoma (LC)	High-expression	Oncogene	proliferation, migration, invasion, glycolysis, EMT and tumorigenicity	PI3K, p-AKT and total AKT	9,11,13
Lung squamous cell carcinoma (LSCC)	Low-expression				9
Hepatocellular carcinoma (HCC)	High-expression	Oncogene	immune evasion	STAT1, IFNGR1, ACTA2, PD-L1	14
			Stemness, interaction with the mitochondria, metabolic activities, metastasis, cell growth	GSK3β, β-catenin	17
			growth and proliferation, glucose metabolism, mitochondrial function, tricarboxylic acid (TCA) cycle, stemness	cyclins/CDKs, GLUT4	16
			tumor diameter, proliferation and migration	β-Catenin and c-Myc	15
Cholangiocarcinoma	High-expression	Oncogene			16
Gastric cancer (GC)	High-expression	Oncogene	EMT, invasion and metastasis, drug resistance, lipogenesis	G3BP1, PRKDC	23
			viability, colony formation, migration, invasion abilities, tumor size		22
			glycolysis, tumorigenesis, and EMT, drug resistance	NF-κB	25
			proliferation, oxygen consumption and decreased glycolytic protein expression, glucose absorption, lactate production, ATP level, and extracellular acidification ratio		24
Colorectal cancer (CC)	High-expression	Oncogene	promoted proliferation, metastasis and glycolysis	circVMP1	19

**Table 2:** Clinical significance of HKDC1 in various cancers

Cancer type	Clinicopathological features	References
Lung cancer	shorter overall survival	11
Lung adenocarcinoma	histologic differentiation, pathological N stage, shorter overall survival	9
Hepatocellular carcinoma	progression free survival	14
Hepatocellular carcinoma	tumor stages, shorter overall survival	16
Hepatocellular carcinoma	shorter overall survival	15
Colorectal cancer	higher overall survival	31
Gastric cancer (GC)	shorter overall survival	22,23,25
Pancreatic cancer	shorter overall survival	26
